# The WHAM Study: Socio-Emotional Well-being Effects of Hearing Aid Use and Mediation Through Improved Hearing Ability

**DOI:** 10.1097/AUD.0000000000001700

**Published:** 2025-07-25

**Authors:** Lotte A. Jansen, Marieke F. van Wier, Birgit I. Lissenberg-Witte, Cas Smits, Sophia E. Kramer

**Affiliations:** 1 Section Ear and Hearing, Department of Otolaryngology-Head and Neck Surgery, Amsterdam UMC Location Vrije Universiteit Amsterdam, Amsterdam, the Netherlands; 2 Quality of Care, Amsterdam Public Health Research Institute, Amsterdam, the Netherlands; 3 Department of Epidemiology and Data Science, Amsterdam UMC Location Vrije Universiteit Amsterdam, Amsterdam, the Netherlands; 4 Section Ear and Hearing, Department of Otolaryngology-Head and Neck Surgery, Amsterdam UMC Location Universiteit van Amsterdam, Amsterdam, the Netherlands.

**Keywords:** Duration, Frequency, Hearing aid uptake, Well-being

## Abstract

**Objectives:**

Hearing impairment can negatively impact socio-emotional well-being. While hearing aids (HA) may improve hearing ability, communication, social participation, and emotional well-being, longitudinal studies are scarce and evidence quality is low. This longitudinal study examines the associations between (research question [RQ] 1) HA uptake and socio-emotional well-being, mediation by self-perceived hearing disability, and differences between subgroups, (RQ2) frequency of HA use (daily number of hours) and socio-emotional well-being, and (RQ3) duration of HA use (years of use) and socio-emotional well-being.

**Design:**

Data from October 2006 to January 2024 from the Netherlands Longitudinal Study on Hearing were used for this study. Every 5 yrs, participants were invited to complete an online digits-in-noise hearing test and survey, which included variables on HA use, psychosocial health, tinnitus, hyperacusis, and self-perceived hearing disability. For RQs 1 and 2, cumulative data from three 5-yr intervals (baseline [T0] to 5-yr follow-up [T1], T1–T2, and T2–T3) was compiled, based on eligibility for a HA at the beginning of the studied time interval but not using it at that time and either reporting HA use (HA uptake) or no HA use (no HA uptake) at follow-up and frequency of use at follow-up. Differences between those who adopted a HA versus those who did not were examined while controlling for pre-(non)uptake socio-emotional outcomes. After applying exclusion criteria, the final samples included n = 281 unique participants for RQ1 and n = 280 for RQ2. For RQ3, participants with 5, 10, or 15 yrs of HA use were identified and analyzed to assess the impact of long-term use, with n = 180 unique participants in the final dataset. Outcomes assessed for each RQ were depression, anxiety, distress, somatization, social loneliness, emotional loneliness, and total loneliness. Gamma regression models with generalized estimating equations were performed to analyze all RQs.

**Results:**

Approximately 87% of participants were ≤65 yrs of age at T0. Among individuals without tinnitus, HA uptake was significantly associated with lower depression scores (*p <* 0.05). Among those aged >65 yrs, HA uptake was significantly associated with lower total loneliness scores. No significant associations were found between HA uptake and anxiety, somatization, distress, and emotional loneliness. Self-perceived hearing disability did not mediate the relationship between HA uptake and socio-emotional well-being outcomes. No significant associations between the duration of HA use and socio-emotional well-being outcomes were found. Frequency of HA use was not significantly associated with any outcome except somatization, where using a HA for 1 to 4 hrs per day was significantly associated with lower somatization scores.

**Conclusions:**

This longitudinal study contributes valuable evidence to the growing body of research on the psychosocial benefits of HAs, highlighting both the potential and the limitations of device use in improving well-being. Results suggest that audiologists might consider integrating psychosocial support as part of a comprehensive treatment approach beyond simply recommending HA adoption or increased usage.

## INTRODUCTION

Hearing impairment can negatively impact emotional well-being (Nachtegaal et al. 2009; Heffernan et al. 2016; Holman et al. 2019). It has been associated with mental health symptoms such as anxiety (Contrera et al. 2016; Jayakody et al. 2018), depression (Nachtegaal et al. 2009; Lawrence et al. 2020), distress and somatization (Nachtegaal et al. 2009). Hearing impairment can also make communication more challenging as it demands more listening effort, resulting in negative emotions such as frustration (Heffernan et al. 2016; Bennett et al. 2021). Individuals who feel frustrated during challenging listening situations can feel less motivated to socially participate in these situations (Holman et al. 2019) and consequently socially isolate. Indeed, the current literature indicates that individuals with hearing impairment have a higher likelihood of experiencing loneliness and social isolation (Nachtegaal et al. 2009; Pronk et al. 2011; Shukla et al. 2020). Effective rehabilitation interventions are vital to mitigate these adverse outcomes and support overall well-being. The present study aims to examine the impact of hearing rehabilitation on social and emotional well-being.

Hearing aids (HA) are one common form of hearing rehabilitation and have the potential to improve several domains of hearing ability such as speech understanding (Ferguson et al. 2017; de Ronde-Brons et al. 2019), which in turn may facilitate communication and increase social participation (Oosthuizen et al. 2022) and feelings of social connectedness (Ferguson et al. 2017), decrease loneliness (Weinstein et al. 2016), and improve confidence in communication abilities (Oosthuizen et al. 2022). Research on the effects of HAs on loneliness generally shows mixed results showing that users experience short-term declines in loneliness post-fitting (Weinstein et al. 2016) or experience no benefit on loneliness (Pronk et al. 2011; Applebaum et al. 2019). Current evidence also suggests that HA use may be associated with lower psychological distress (Bigelow et al. 2020), depression (Nkyekyer et al. 2019; Wells et al. 2020; Tsimpida et al. 2022), and improved quality of life (Stark & Hickson 2004). While most of these studies have cross-sectional or pre-post designs, which are not suitable for drawing causal conclusions (Munro et al. 2021), one randomized controlled trial (RCT) showed that HAs significantly improved depressive symptoms after 4 wks of use (Marques et al. 2022). Some qualitative studies have also reported improvements in positive emotions (Holman et al. 2023) and psychosocial functioning (Oosthuizen et al. 2022) among HA users. A clear or causal relationship between HA use and improvement in well-being outcomes remains to be established.

It is also unclear whether certain subgroups (e.g., older adults, women, etc.) experience different effects of HA use on well-being. Older adults, often experiencing hearing loss and/or social isolation, may benefit from HAs in improving social participation. Gender differences in patterns of HA use (Staehelin et al. 2011) and in the effect of hearing impairment on well-being (Harada et al. 2008) have been reported, warranting further exploration into how HA uptake and use impacts men and women differently. In addition, both tinnitus and hyperacusis, often co-occurring with hearing impairment and linked to poorer mental health (Sacchetto et al. 2022; Hackenberg et al. 2023), warrant investigation into how HAs affect well-being in these subgroups. This is important for ultimately tailoring rehabilitation strategies to meet the specific needs of these populations and maximizing the benefits of HAs on well-being.

In addition, Gopinath et al. (2012) found that, compared with the severity of hearing loss, perceived hearing ability was a strong predictor of changes in quality of life among older adults. Similarly, Knudsen et al. (2010) showed that perceived hearing disability is a more important factor in the success of hearing rehabilitation than objective hearing loss measures (pure-tone audiometry). Yet it is not clear whether the positive effects of HAs on well-being can be explained by improved self-perceived hearing disability following HA adoption. More research using longitudinal designs is needed to clarify the causal pathways between HA use and well-being, while also exploring subgroup differences and the mediating role of perceived hearing disability.

Furthermore, it is not yet clear how much time is needed after the initial fitting of an HA to yield benefits in socio-emotional well-being. Current evidence shows mostly short-term benefits of HA use (Tesch-Römer 1997; Stark & Hickson 2004; Contrera et al. 2016; Ferguson et al. 2017; Borre et al. 2023) while long-term (>1 yr) improvements are generally lacking. Moreover, individuals may own an HA but not necessarily use them consistently or at all. One meta-analysis of 21 studies showed that 38% of individuals with HAs do not use them (Marcos-Alonso et al. 2023). However, for those who do use their HAs, the more hours used per day, the higher the quality of life reported among both first-time and experienced users (Wolff et al. 2024). More research is needed to understand the roles of duration and frequency of use on socio-emotional well-being.

While ideally an RCT with a longer timeframe (>1 yr) would be preferred to examine the effects of HAs on socio-emotional well-being, this would not adhere to ethical standards given the negative effects of hearing loss and current evidence for beneficial short-term outcomes of HA use (Munro et al. 2021; Marques et al. 2022). Therefore, we applied a longitudinal observational study design using principles of causal inference to emulate an RCT (Kutcher et al. 2021). In this design, we included participants with disabling hearing impairment who are non-HA users and are followed over time. Long-term outcomes were compared between those who adopted an HA with those who did not. Statistical methods were used to account for the nonrandomized design and differences in group characteristics. The present study aims to fill in the current gaps in the literature using this study design and examine the research questions (RQs) presented below:

1. What is the association between HA adoption and social and emotional well-being as compared to not adopting an HA?
  - Does this association differ for the following subgroups: young-middle age adults (≤65 yrs) versus older adults (>65 yrs); adults with tinnitus versus no tinnitus; adults with hyperacusis versus no hyperacusis; men versus women?
  - Is this association mediated by self-reported hearing disability? Which of the five domains of hearing ability (speech intelligibility in noise, intelligibility in quiet, auditory localization, distinguishing sounds, and detection of sounds) are responsible for this effect?
2. What is the association between the frequency of HA use (daily number of hours) and well-being outcomes?
3. What is the association between the duration of HA use (number of years) and well-being outcomes as compared with not adopting an HA for the same number of years?

## MATERIALS AND METHODS

### Study Design and Setting

Quantitative data from the Netherlands Longitudinal Study on Hearing (NL-SH) from October 2006 to January 2024 was analyzed. The NL-SH is an online longitudinal cohort study that examines associations between hearing ability and various aspects of life, including psychosocial health. Data collection started in 2006 and enrollment is currently ongoing. Adults with or without hearing impairment and between 18 and 70 yrs of age at enrollment are eligible to participate. Participants complete an online survey and speech-in-noise test every 5 yrs (baseline: T0, 5-yr: T1, 10-yr: T2, and 15-yr: T3 follow-up rounds) or until voluntary exit from the study. More details regarding the NL-SH protocol can be found in van Wier et al. (2023). This protocol was approved by the institutional review board (Medical Ethics Committee). As of 2016, the study has been retrospectively deemed outside the scope of the Medical Research Involving Human Subjects Act, requiring no additional ethical review.

### Sample Population

#### RQ1, 1(a), and 1(b)

For RQs 1, 1(a), and 1(b), all 3087 NL-SH participants were assessed for eligibility. Participants were excluded if they reported cochlear implant use at any measurement round, reported having hearing loss before the age of 18 yrs (as this may affect emotional well-being differently than at a later age), and reported using an HA at T1 but not at T2 (which increases the unreliability of HA use response data). In addition, participants who either (1) self-reported having some type of hearing disability but had a speech reception threshold in noise (SRTn) score of <−5.5 dB signal to noise ratio (SNR), or (2) self-reported having normal hearing regardless of SRTn score and did not report using an HA at the follow-up measurement rounds (T1, T2, or T3), were excluded. An SRTn cut-off score of ≥−5.5 dB SNR was used to determine eligibility for an HA as −5.5 dB SNR corresponds to a pure-tone average of 35 dB at 1, 2, and 4 kHz, which is the threshold for HA reimbursement in the Netherlands (Smits et al. 2004). Two hundred and sixteen participants met the criteria at T0 to T1, 75 participants at T1 to T2, and 35 at T2 to T3. Note that participants in the final T0 to T1 sample could also be included in the T1 to T2 or T2 to T3 samples (i.e., participants who did not adopt an HA in T0 to T1 may have adopted one in T1 to T2 or T2 to T3). The number of participants who overlapped across samples was 45 (SM1, Figure 1, Supplemental Digital Content, https://links.lww.com/EANDH/B686). In total, 281 unique participants were included in analyses for these RQs.

#### RQ2

For RQ2, the same exclusion criteria from RQ1 were applied. Participants were further excluded if they did not provide any data on the frequency of HA use (SM1, Figure 2, Supplemental Digital Content, https://links.lww.com/EANDH/B686). In total, 280 unique participants were included in analyses for RQ2, with 44 participants overlapping across samples.

#### RQ3

For RQ3, the same exclusion criteria from RQ1 were applied. In addition, participants who reported a duration of HA use that was inconsistent with the time at which they first reported using an HA (i.e., if they adopted an HA at T1 but reported having used this HA for >5 yrs), were excluded (SM1, Figure 3, Supplemental Digital Content, https://links.lww.com/EANDH/B686). In total 180 unique participants were included in these analyses, with 80 overlapping across different durations of use (5, 10, and 15 yrs of HA use/nonuse).

### Measures

#### Dependent Variables

##### Emotional well-being

The Four-Dimensional Symptom Questionnaire was used to assess emotional well-being. This questionnaire comprises 50 items reflecting psychological symptoms experienced during the past 7 days, assessed using 5-point Likert scale from “no” (0) to “very often or constantly” (4) (Terluin et al. 2006). It measures 4 subscales of psychological health, namely depression (0 to 12), anxiety (0 to 24), distress (0 to 32), and somatization (0 to 32). Likert responses were recoded (“no” = 0, “sometimes” = 1, “regularly” = 2, “often” = 2, “very often or constantly” = 2) to calculate sum scores for each subscale (Terluin et al. 2006). If more than two depression, four anxiety, six distress, or six somatization items were missing, sum scores for that subscale were not calculated.

##### Social well-being

The de Jong-Gierveld Scale was used to assess social well-being. It contains 11 items measuring an individual’s subjective experiences/feelings of emotional and social loneliness. Social and emotional loneliness scores were calculated according to the scale’s manual (de Jong-Gierveld 1999) and could range from 0 to 5 and 0 to 6, respectively. If one item was missing, the score was not calculated. The sum of the emotional and social loneliness scores created the total loneliness score and could range from 0 to 11. If more than one item was missing, the total loneliness sum score was not calculated.

For all dependent variables, higher scores indicate stronger symptoms/poorer emotional and social well-being.

### Independent Variables

### RQ1, 1(a), and 1(b)

SM2, Figure 1, Supplemental Digital Content, https://links.lww.com/EANDH/B686, represents the longitudinal timeline of the study and the independent variables measured at each measurement round for RQ’s 1, 1(a) and 1(b). Only participants who reported not using HAs were included at T0 and were observed whether they adopted an HA at later measurement rounds. HA uptake was defined as a dichotomous variable at T1, T2, and T3 and measured using the survey question “Do you use a hearing aid?” (“Yes” or “No”). A participant was given a value of 1 if they were eligible for an HA or reported normal hearing at the prior measurement round, and answered “Yes” at the follow-up measurement round. A value of 0 was given if a participant answered “No” to “Do you use a hearing aid?” at follow-up but was eligible for an HA during the prior measurement round. HA uptake was measured at 5-yr intervals (T0 to T1, T1 to T2, and T2 to T3).

To assess subgroup effects for RQ1(a), a dichotomous variable for age group was created (≤65, >65 yrs). Tinnitus was measured with the survey question, “Do you suffer from ringing in the ears (tinnitus)?” (“Yes”/“No”). Hyperacusis was measured with, “Do you think that loud noises bother you more than they do other people?” (“Yes”/“No”). Sex options were “female” or “male.” For RQ1(b), self-perceived hearing disability was assessed using the Amsterdam Inventory for Auditory Disability and Handicap (AIADH), which contains 28 items that measure self-perceived hearing disability in everyday listening situations (Kramer et al. 1995). Responses are on a four-point Likert Scale (“almost never” (3) to “almost always” (0)) and higher scores reflect higher disability. The AIADH measures five hearing disability factors: distinguishing sounds, auditory localization, detection of sounds, intelligibility in noise, and intelligibility in quiet. A total mean score was calculated using factor mean scores, with the mean value of other values imputed if one missing value was present in a factor.

### RQ2

Frequency of HA use was measured as a categorical independent variable at T1, T2, and T3 using the International Outcome Inventory for Hearing Aids question “On average, how many hours per day during the past 2 wks did you use your hearing aid?” (Kramer et al. 2002). Due to insufficient cases (<20) in the “<1 hour” category, International Outcome Inventory for Hearing Aids categories were reorganized and merged to: “not at all or <1 hour” (0), “1 to 4 hours” (1), and “>4 hours” (2). The first category (0) represented the reference category for analyses. In addition, non-HA users (and thus, no frequency of use) were added to this reference category.

### RQ3

SM2, Figure 2, Supplemental Digital Content, https://links.lww.com/EANDH/B686, represents how the duration of HA use was measured and defined. Only participants who reported not using an HA were included at T0. If a participant answered “Yes” to “Do you use a hearing aid?” at T1, they were deemed to have used an HA for >0 and ≤5 yrs (between T0 and T1). If they answered “Yes” at T2, they were deemed to have used an HA for >0 and ≤5 yrs (T1 to T2) and not used an HA for 5 yrs (between T0 and T1). Similarly, if a participant answered “Yes” at T3, they were deemed to have used an HA for >0 and ≤5 yrs (between T2 and T3) and not used an HA for 10 yrs (between T0 and T2). After a participant initially reports using or not using an HA (T1 or T2), depending on how long they remain in the study, they may use or not use the HA for 5 or 10 yrs. These values of HA use and nonuse were compiled to create the independent variable, time (5 or 10 yrs), representing the duration of HA use or nonuse. Too few participants (n = 26) reported a duration of 15 yrs for generalized estimating equations (GEE) models to be reliable, so this duration was ultimately not included in the analyses. An interaction term between HA use and time was entered into the statistical model to assess the effect of duration of HA use/nonuse.

### Confounders and Effect Modifiers

Age group, sex, living situation (“alone”/”with others”), educational level (“low”/“mid”/”high”), employment status (“paid job”/“retired”/“other”), health status (0: poor to 100: excellent), tinnitus, and hyperacusis were assessed for confounding and effect modifying effects. Health status (“Could you indicate between 0 [worst state] and 100 [best state] what your health state today is?” 0 to 100) was categorized into quartiles due to violations of the linearity assumption with the dependent variables. In addition, SRTn scores were assessed for confounding and modification effects. To generate these scores, participants completed the National Hearing Test, an online adaptive speech-in-noise test in which 23-digit triplets are presented against a background of noise. SRTn scores were derived by averaging the SNR of the final 20 triplets (corresponding to 50% intelligibility) (Smits et al. 2004).

Because research has shown associations between coping behaviors and better psychosocial health (Warringa et al. 2020), six subscales of the Communication Profile for the Hearing Impaired, which measure six coping behaviors (maladaptive behavior, stress and withdrawal, self-acceptance, verbal strategies, nonverbal strategies, and acceptance of loss), were assessed for confounding effects. The Communication Profile for the Hearing Impaired contains 35 items with responses on a 5-point Likert scale (1: almost never to 5: almost always, or 1: strongly disagree to 5: strongly agree). Scores for all scales, except for verbal strategies and nonverbal strategies, were reversed so that lower scores reflected potential coping difficulties. Higher scores for all items reflect better coping behaviors. Mean scores for each subscale were created (Mokkink et al. 2009).

Baseline depression, anxiety, distress, somatization, total loneliness, emotional loneliness, and social loneliness scores were included in all statistical models to correct for baseline differences between groups. Baseline scores correspond to baseline values of the observation period (i.e., T0 for T0 to T1, T1 for T1 to T2, and T2 for T2 to T3). For all RQs, age group, sex, and education level were also measured at baseline, while the remaining variables were measured at the follow-up measurement round(s).

### Statistical Analyses

Descriptive statistics were produced for all dependent, independent, and sociodemographic variables. Longitudinal regressions for all RQs were conducted using GEE with an exchangeable correlation structure and a Gamma distribution, given the right-skewed distributions of the outcome variables. Depending on model fit, a log link or inverse link was used. For outcome variables with a minimum sum score of 0, 0.1 was added to avoid zero values, as Gamma regressions require positive scores. Confounding tests for all RQs involved (i) testing for significant associations between confounders and independent and dependent variables (*p <* 0.1) and (ii) checking for ≥10% change in *β* coefficient after adding the confounder to the model (Ter Wee & Lissenberg-Witte 2019). An HA uptake value of 0 was the reference group for all RQ1 analyses. For RQ1(a), interaction terms for subgroups of interest (age group, tinnitus, hyperacusis, sex) and HA uptake were added to the model in RQ1 to test for effect modification. If significant (*p <* 0.1), regression results were reported per stratum of subgroup. For RQ1(b), mediation analyses using R package GEEmediate (Nevo et al. 2017) assessed natural direct/indirect effects and proportion mediated for AIADH scores.

For RQ2, frequency of HA use was the main independent variable in the model. The reference group (0) consisted of individuals who were nonusers, had an HA but did not use it, or used an HA for <1 hr per day during the past 2 wk. Variables described in the previous section were tested for confounding and effect modification. For RQ3, each GEE model included an interaction effect between HA use and time. The reference groups consisted of non-HA users (HA use = 0) and a duration of >0 and ≤5 yrs (time = 5 yrs). Variables described in the previous section were tested for confounding and effect modification.

All GEE analyses produced *β* coefficients and 95% confidence intervals (*p <* 0.05). Estimated marginal means (EMMs) were produced to facilitate the interpretation of these statistics. Analyses were conducted using IBM SPSS Statistics 28 and RStudio 4.3.2.

## RESULTS

### Participant Characteristics

Table 1 shows descriptive data for sociodemographic and dependent variables at measurement rounds T0, T1, and T2. A total of N = 281 unique participants were included for RQ1, with N = 45 overlapping in T0, T1, and T2 samples (SM1, Figure 1, Supplemental Digital Content, https://links.lww.com/EANDH/B686). Participants were predominantly female (ranging from 48.6 to 60.0%), highly educated (48.6 to 62.9%), and lived with others (74.1 to 76.5%). The average SRTn score ranged between −3.0 and −4.0 dB SNR. Of the initial sample, 51.9% adopted an HA at T1, and over 50% of participants in the subsequent follow-up periods adopted an HA.

**TABLE 1.** Participant statistics for RQ1

	T0–T1* (N = 216)	T1–T2* (N = 75)	T2–T3* (N = 35)
Sociodemographics			
Age group (yr), n (%)			
≤65	187 (86.6)	53 (70.7)	19 (54.3)
>65	29 (13.4)	22 (29.3)	16 (45.7)
n (%)	216 (100.0)	75 (100.0)	35 (100.0)
Sex, n (%)			
Female	120 (55.6)	45 (60.0)	17 (48.6)
Male	96 (44.4)	30 (40.0)	18 (51.4)
n (%)	216 (100.0)	75 (100.0)	35 (100.0)
Living situation, n (%)			
Alone	54 (25.0)	18 (24.0)	8 (23.5)
With others	160 (74.1)	57 (76.0)	26 (76.5)
n (%)	214 (99.1)	75 (100.0)	34 (97.1)
Education level, n (%)			
Low	44 (20.6)	13 (17.6)	2 (5.8)
Mid	62 (29.0)	25 (33.7)	11 (31.4)
High	108 (50.5)	36 (48.6)	22 (62.9)
n (%)	214 (99.1)	74 (98.7)	35 (100.0)
Employment status, n (%)			
Paid job	89 (41.2)	19 (25.3)	6 (17.1)
Retired	69 (31.9)	35 (46.7)	18 (51.4)
Other	58 (26.9)	21 (28.0)	11 (31.4)
n (%)	216 (100.0)	75 (100.0)	35 (100.0)
Health status, M (SD)	69.9 (25.1)	70.4 (24.3)	71.3 (23.3)
n (%)	207 (95.8)	75 (100.0)	34 (97.1)
Tinnitus, n (%)	133 (64.6)	46 (63.0)	19 (59.4)
n (%)	206 (95.4)	73 (97.3)	32 (91.4)
Hyperacusis, n (%)	95 (52.8)	36 (56.2)	11 (39.2)
n (%)	180 (83.3)	64 (85.3)	28 (80.0)
SRTn (dB SNR), M (SD)	−3.0 (3.3)	−3.8 (3.2)	−4.0 (3.1)
n (%)	195 (90.2)	69 (92.0)	35 (100.0)
Hearing aid uptake, n (%)			
Yes	112 (51.9)	38 (50.7)	23 (65.7)
No	104 (48.1)	37 (49.3)	12 (34.3)
n (%)	216 (100.0)	75 (100.0)	35 (100.0)
Coping behaviors			
Maladaptive coping (0–5), M (SD)	4.2 (0.7)	4.3 (0.6)	4.4 (0.4)
n (%)	216 (100.0)	75 (100.0)	35 (100.0)
Nonverbal strategies (0–5), M (SD)	3.3 (0.9)	3.3 (1.0)	2.9 (0.9)
n (%)	216 (100.0)	75 (100.0)	35 (100.0)
Verbal strategies (0–5), M (SD)	2.5 (0.8)	2.4 (0.7)	2.2 (0.8)
n (%)	216 (100.0)	75 (100.0)	35 (100.0)
Self-acceptance (0–5), MD (SD)	4.1 (0.86)	4.2 (0.9)	4.1 (0.9)
n (%)	215 (99.5)	75 (100.0)	35 (100.0)
Stress & withdrawal (0–5), M (SD)	3.3 (0.9)	3.4 (0.8)	3.8 (0.8)
n (%)	215 (99.5)	75 (100.0)	34 (97.1)
Acceptance of loss, M (SD)	3.6 (0.90)	3.6 (0.95)	3.8 (1.00)
n (%)	215 (99.5)	75 (100.0)	34 (97.1)
Self-perceived hearing disability			
Distinguishing sounds (0–3), M (SD)	0.7 (0.56)	0.7 (0.54)	0.5 (0.44)
n (%)	214 (99.1)	75 (100.0)	34 (97.1)
Auditory localization (0–3), M (SD)	1.5 (0.8)	1.4 (0.8)	1.1 (0.8)
n (%)	215 (99.5)	75 (100.0)	34 (97.1)
Detection of sounds (0–3), M (SD)	0.9 (0.5)	0.8 (0.5)	0.7 (0.5)
n (%)	215 (99.5)	75 (100.0)	34 (97.1)
Intelligibility in noise (0–3), M (SD)	1.7 (0.6)	1.6 (0.6)	1.0 (0.6)
n (%)	215 (99.5)	75 (100.0)	34 (97.1)
Intelligibility in quiet (0–3), M (SD)	1.2 (0.6)	1.1 (0.6)	1.4 (0.7)
n (%)	215 (99.5)	75 (100.0)	34 (97.1)
Emotional well-being			
Depression (0–12), median (IQR)	0.0 (1.0)	0.0 (1.0)	0.0 (0.0)
n (%)	210 (97.2)	75 (100.0)	34 (97.1)
Baseline depression, median (IQR)	0.0 (1.0)	0.0 (1.0)	0.0 (1.0)
n (%)	216 (100.0)	74 (98.7)	35 (100.0)
Anxiety (0–24), median (IQR)	1.0 (2.0)	1.0 (2.0)	1.0 (2.5)
n (%)	210 (97.2)	75 (100.0)	34 (97.1)
Baseline anxiety, median (IQR)	1.0 (2.8)	1.0 (2.0)	0.0 (2.0)
n (%)	216 (100.0)	74 (98.7)	35 (100.0)
Distress (0–32), median (IQR)	6.0 (9.0)	7.0 (9.0)	6.0 (10.0)
n (%)	210 (97.2)	75 (100.0)	34 (97.1)
Baseline distress, median (IQR)	6.0 (8.0)	6.0 (11.0)	4.0 (9.0)
n (%)	216 (100.0)	74 (98.7)	35 (100.0)
Somatization (0–32), median (IQR)	6.0 (8.0)	5.0 (8.0)	6.0 (7.3)
n (%)	211 (97.7)	75 (100.0)	34 (97.1)
Baseline somatization, median (IQR)	7.0 (7.0)	6.0 (6.0)	6.0 (7.0)
n (%)	216 (100.0)	74 (98.7)	35 (100.0)
Social well-being			
Total loneliness (0–11), median (IQR)	2.0 (5.0)	3.0 (6.0)	3.0 (6.0)
n (%)	207 (95.8)	75 (100.0)	34 (97.1)
Baseline loneliness, median (IQR)	2.0 (6.0)	3.0 (6.0)	3.0 (3.0)
n (%)	215 (99.5)	74 (98.7)	35 (100.0)
Emotional loneliness (0–6), median (IQR)	1.0 (3.0)	1.0 (4.0)	1.0 (4.0)
n (%)	207 (95.8)	75 (100.0)	34 (97.1)
Baseline emotional loneliness, median (IQR)	1.0 (3.0)	1.0 (3.0)	1.0 (2.0)
n (%)	215 (99.5)	74 (98.7)	35 (100.0)
Social loneliness (0–5), median (SD)	1.0 (3.0)	2.0 (4.0)	2.0 (3.3)
n (%)	207 (95.8)	75 (100.0)	34 (97.1)
Baseline social loneliness, median (IQR)	1.0 (3.0)	2.0 (4.0)	1.0 (3.0)
n (%)	215 (99.5)	74 (98.7)	35 (100.0)

Statistics shown pertain to RQ1. All variables are measured at follow-up (T1, T2, or T3) except variables measured at baseline: baseline outcome scores, age, sex, education level.

* Samples correspond to 5-yr NL-SH measurement rounds after exclusion criteria were applied (SM1, Figure 1, Supplemental Digital Content, https://links.lww.com/EANDH/B686). n = 45 participants overlap across the samples.

IQR (difference between 75th and 25th percentiles), interquartile range; M, mean; n, sample size; SNR, signal to noise ratio; SRTn, speech reception threshold in noise.

### RQ1 and 1(a): Five-Year HA Uptake and Subgroup Effects

#### Emotional Well-being

Table 2 shows statistical results for emotional well-being outcomes. Analyses showed that HA uptake interacted significantly with tinnitus on depression (*p* = 0.011). Among those without tinnitus, HA uptake was significantly associated with lower depression scores (*p <* 0.05). Interactions between HA uptake and hyperacusis on distress (*p* = 0.037) and anxiety (*p* = 0.093) were also significant. After stratifying by hyperacusis, HA uptake was not significantly associated with distress nor anxiety. HA uptake was not associated with somatization. No subgroup effects were found for somatization either (*p* > 0.1). EMMs for each outcome are shown in SM3, Table 1, Supplemental Digital Content, https://links.lww.com/EANDH/B686.

**TABLE 2.** GEE results for the associations between HA uptake and emotional well-being outcomes

	Depression		Anxiety		Distress*		Somatization†	
	*b* (95% CI)	*p*	*b* (95% CI)	*p*	*b* (95% CI)	*p*	*b* (95% CI)	*p*
Subgroup								
Tinnitus	0.19 (−0.39 to 0.77)	0.52						
No tinnitus	−1.19 (−1.82 to −0.55)	<0.001‡						
Hyperacusis			0.11 (−0.38 to 0.60)	0.67	−0.02 (−0.04 to 0.00)	0.063		
No hyperacusis			−0.48 (−0.98 to 0.03)	0.065	0.02 (−0.02 to 0.06)	0.27		
Total sample							−0.15 (−0.33 to 0.04)	0.12

All models are adjusted for baseline outcome scores and, if indicated, additional confounders. Only subgroup effects for which significant interaction effects with HA uptake were found are presented.

* Beta estimates represent change in the inverse of the expected value of the outcome variable.

† Additionally adjusted for hyperacusis, employment status, and stress and withdrawal coping score.

‡ *p* < 0.05: statistically significant finding.

*b*, beta coefficient; CI, confidence interval; GEE, generalized estimated equations; HA, hearing aid.

#### Social Well-being

Table 3 shows statistical results for social well-being outcomes. HA uptake was not significantly associated with emotional loneliness. The age group (≤65 versus > 65 yrs) significantly interacted with HA uptake for both total loneliness (*p* = 0.049) and social loneliness (*p* = 0.017). Among those aged >65 yrs, HA uptake was significantly associated with lower total loneliness scores (*p <* 0.05), but was not associated with social loneliness in either age group. A significant interaction was also found for HA uptake and tinnitus on social loneliness (*p* = 0.082), although after stratification, HA uptake was not associated with social loneliness. EMMs for each outcome are shown in SM3, Table 1, Supplemental Digital Content, https://links.lww.com/EANDH/B686.

**TABLE 3.** GEE results for the associations between HA uptake and social well-being outcomes

	Total Loneliness		Social Loneliness		Emotional Loneliness*	
	*b* (95% CI)	*p*	*b* (95% CI)	*p*	*b* (95% CI)	*p*
Subgroup						
≤65 yrs	0.02 (−0.02 to 0.06)	0.36	−0.03 (−0.11 to 0.06)	0.51		
>65 yrs	0.02 (0.02–0.03)	<0.001†	0.000 (0.00–0.00)	0.44		
Tinnitus			−0.04 (−0.14 to 0.05)	0.37		
No tinnitus			0.11 (−0.02 to 0.23)	0.10		
Total sample					0.02 (−0.06 to 0.10)	0.61

All models are adjusted for baseline outcome scores and, if indicated, additional confounders. Only subgroup effects for which significant interaction effects with HA uptake were found are presented. Beta estimates are reported for all models and represent change in the inverse of the expected value of the outcome variable.

* Additionally adjusted for education level, living situation, and stress and withdrawal coping score.

† *p* < 0.05: statistically significant finding.

*b*, beta coefficient; CI, confidence interval; GEE, generalized estimated equations; HA, hearing aid.

### RQ1(b): Mediation Through Self-Perceived Hearing Disability

Mediation analyses were conducted to examine the effect of HA uptake on emotional and social well-being outcomes, with each AIADH factor and total score as a mediator. SM3, Table 2, Supplemental Digital Content, https://links.lww.com/EANDH/B686, shows the natural indirect and direct effects tested for the mediators. Results show that the natural indirect effects of HA uptake on all well-being outcomes through each AIADH factor and the total AIADH score were not statistically significant (*p* > 0.1), indicating self-perceived hearing disability does not mediate the relationship between HA uptake and socio-emotional well-being.

### RQ2: Frequency of HA Use

#### Emotional Well-being

Among sample participants, 177 were non-HA users or owned an HA but used it for <1 hr per day. Twenty-two participants used an HA for 1 to 4 hr, and 128 used it for >4 hr per day. Table 4 shows statistical results for emotional well-being outcomes. Adjusted results showed that the frequency of HA use was not significantly associated with depression, anxiety, or distress. Using an HA for 1 to 4 hr per day was significantly associated with lower somatization scores (*p <* 0.05). SM3, Table 4, Supplemental Digital Content, https://links.lww.com/EANDH/B686, shows EMMs for each outcome.

**TABLE 4.** GEE results for the associations between frequency of HA use and emotional well-being outcomes

	Depression		Anxiety		Distress		Somatization	
Frequency of HA Use	*b* (95% CI)	*p*	*b* (95% CI)	*p*	*b* (95% CI)	*p*	*b* (95% CI)	*p*
1–4 hr per day	0.20 (−0.61 to 1.00)	0.64	−0.26 (−0.79 to 0.27)	0.33	−0.12 (−0.48 to 0.24)	0.52	−0.38 (−0.70 to −0.06)	0.020*
>4 hr per day	−0.04 (−0.58 to 0.49)	0.88	−0.10 (−0.48 to 0.28)	0.60	−0.05 (−0.25 to 0.14)	0.59	−0.18 (−0.37 to 0.01)	0.060

All models are adjusted for baseline outcome scores and, if indicated, additional confounders.

* *p* < 0.05: statistically significant finding.

*b*, beta coefficient; CI, confidence interval; GEE, generalized estimated equations; HA, hearing aid.

#### Social Well-being

The same sample was used for social well-being outcomes. SM3, Table 3, Supplemental Digital Content, https://links.lww.com/EANDH/B686, shows statistical results for social well-being outcomes. Adjusted results showed that the frequency of HA use was not significantly associated with total, social, or emotional loneliness scores. SM3, Table 4, Supplemental Digital Content, https://links.lww.com/EANDH/B686, shows EMMs for each outcome.

### RQ3: Duration of HA Use

#### Emotional Well-being

SM3, Table 5, Supplemental Digital Content, https://links.lww.com/EANDH/B686, shows results for emotional well-being outcomes. Duration of HA use was not significantly associated with any emotional well-being outcome (*p >* 0.05), indicating that the change in the mean emotional well-being outcomes from using an HA from 0 to 5 yrs to 5 to 10 yrs was not statistically different from those who did not use an HA for this duration. SM3, Table 7, Supplemental Digital Content, https://links.lww.com/EANDH/B686, shows EMMs for each outcome.

#### Social Well-being

SM3, Table 6, Supplemental Digital Content, https://links.lww.com/EANDH/B686, shows results for social well-being outcomes. Duration of HA use was not significantly associated with any social well-being outcome (*p >* 0.05). SM3, Table 7, Supplemental Digital Content, https://links.lww.com/EANDH/B686, shows EMMs for each outcome.

## DISCUSSION

### RQ1: HA Uptake and Well-being Outcomes

Individuals with hearing impairment have identified communication, reduction in listening effort and fatigue, employment, social connectedness, emotional well-being, and coping with hearing loss and its social stigma as priorities (Granberg et al. 2014; Vas et al. 2017; Dixon et al. 2020). Given the negative effects of hearing loss on these priorities, HAs may be a solution. The first aim of this study was to explore the long-term effects of HA uptake on socio-emotional well-being, considering factors such as age group, tinnitus, hyperacusis, and sex, and whether these effects were influenced by self-perceived hearing disability.

HA uptake was significantly associated with lower depression, consistent with previous studies investigating the relationship between HA use and depression (Mulrow et al. 1990; Nkyekyer et al. 2019; Wells et al. 2020; Marques et al. 2022; Tsimpida et al. 2022). Our result is unique in that we specifically examined individuals without tinnitus, suggesting that those with only hearing impairment may benefit more from HAs in terms of depression. Depression may be linked to greater difficulty in adjusting to the challenges of a chronic condition (Geocze et al. 2013) like hearing loss. In contrast, individuals with both tinnitus and hearing impairment may face more complex challenges, with HAs having a less significant impact on depression. Thus, insignificant findings among those with tinnitus could point to a weaker or more complicated relationship with depression. Tinnitus severity was also not taken into account. In addition, while HA uptake and tinnitus interacted on social loneliness, HA uptake was not linked to social loneliness after stratification. Further research is needed to explore these relationships.

Our findings on loneliness contrast with Contrera et al. (2017) and Applebaum et al. (2019), who found no significant improvements in loneliness among HA users in the short term (6, 12 mo) or long term (5 yrs), respectively. However, we observed a small but significant improvement specifically among older adults. Perhaps older adults perceive HAs as aiding communication and supporting existing social relationships, and younger adults less so, although this hypothesis would require further investigation. Although age group also interacted with HA uptake on social loneliness (Table 3), stratified analyses within each age group revealed no statistically significant differences in loneliness between those who adopted a HA versus those who did not, which can occur when either the direction or strength of the association differs between groups, but differences within each group are not strong enough to reach statistical significance. Nevertheless, differences between younger and older adults warrant further investigation.

Results showed no statistically significant differences in anxiety, distress, somatization, or emotional loneliness scores between individuals who adopted an HA versus those who did not. These findings align with Dawes et al. (2015), whose study found no significant differences in mental health over 5 yrs between HA users and non-users, although their study assessed overall mental health rather than separate components, limiting comparability. Similarly, other intervention studies reported similar findings that HAs did not change mental health measures 6- and 3-mo post-fitting, respectively (Tesch-Römer 1997; Stark & Hickson 2004), although these may not hold in a longitudinal context. Although HAs are expected to improve hearing and well-being, our results mirror Pronk et al. (2014), who found that changes (decline) in hearing over time did not significantly affect anxiety, distress, or somatization symptoms. This could suggest that hearing improvements may not lead to improved well-being, although most individuals scored fairly low for the outcomes tested, indicating little to no psychosocial symptoms (Table 1). Further research is needed to explore the long-term effects. Personal (e.g., resilience, personality) or environmental factors may play a larger role in the relationship between HA uptake and well-being than hearing improvements alone. Previous research suggests that social support increases help-seeking behavior and HA adoption (Mahoney et al. 1996; Meyer & Hickson 2012) and the success of hearing interventions (Hickson et al. 2014). Strong support systems may attenuate the beneficial effects of HAs on well-being, as social support can buffer psychological stress. Future studies ought to explore these pathways when examining the impact of HAs on well-being. Moreover, significant interactions between HA uptake and hyperacusis on distress and anxiety, although ultimately showing insignificant associations after stratification, warrant further investigation.

If a relationship between HA uptake and well-being is not direct, perceived hearing disability may mediate this relationship. While we expected HA uptake to reduce self-perceived disability, our results showed no mediation effect. Self-perceived disability was associated with some well-being outcomes, but HA uptake was not significantly associated with a self-perceived hearing disability (SM3, Table 2, Supplemental Digital Content, https://links.lww.com/EANDH/B686). This contrasts with Tavanai et al. (2023), who found that HA users (versus nonusers) reported higher self-perceived handicap, despite similar communication difficulties and PTA results between the two groups. This suggests that HAs may not adequately address subjective hearing challenges, possibly due to social stigma (e.g., ageism), unrealistic expectations, or dissatisfaction with HAs. In addition, the timeframe between HA adoption and improvements in situational hearing is not known and may vary, where an interval of 5 yrs may be too large to see an effect, overlooking either difficult acclimatization, “honeymoon,” and/or plateau phases of HA use. Future research should assess HA effects within smaller time intervals over a long duration and monitor whether and when these effects plateau to better understand the sustainability of their benefits.

### RQ2: Frequency of HA Use

The second aim of this study was to examine the relationship between the frequency of HA use and well-being outcomes. Frequency of HA use was not significantly associated with any well-being outcome except somatization. Those who used an HA for 1 to 4 hr per day had, on average, lower somatization scores than those who used an HA for <1 hr per day or not at all. There was a similar trend in those who used an HA for >4 hr per day, but this failed statistical significance (*p* = 0.06). In that sense, our findings differed from Ho et al. (2020), who found that using an HA >4 hr per day was associated with an early reduction in self-perceived hearing disabilities post-HA fitting, although they did not assess somatization. Somatization involves an inclination to experience physical symptoms or distress that lack a medical explanation, often attributing these sensations to illness and seeking medical care as a result (Lipowski 1988). In our study, using an HA for a few hours per day may reflect user satisfaction, rather than a constant need for hearing support. HAs may also provide reassurance that auditory or health concerns are being addressed, reducing somatization symptoms, possibly due to a placebo effect. Extended use may be associated with additional stressors, particularly in a working population, which could offset the benefits on somatization. Alternatively, extended use might lead to listening-related fatigue or effort, particularly among first-time users, reducing the psychosocial benefits from shorter usage. Although research on HA use and fatigue is inconclusive (Holman et al. 2021), more studies are needed to explore these dynamics and the relationship between HA use and somatization. It is important to note that the frequencies within the categories of the “frequency of use” variable were not evenly distributed, which could potentially impact the validity of the results. Future studies with larger samples would allow for a more detailed examination of HA use patterns across a broader range of frequencies.

### RQ3: Duration of HA Use/Nonuse

The final aim was to examine the relationship between the duration of HA use and well-being outcomes. Results showed no significant differences in well-being outcomes between those who used an HA for 5 or 10 yrs versus those who did not for that same time period. This is a novel finding. Similarly, Dawes et al. (2015) found no significant differences in mental health outcomes at 5- and 11-yr follow-up, although they did not specifically compare the effects of 5-yr HA use versus 10-yr HA use on these outcomes, limiting insights into how the duration of HA use might influence mental health. To our knowledge, no other studies have explored this. Tavanai et al. (2023) also did not find significant associations between duration of use and depression, although the mean duration of use in their sample was 36.25 mo. We hypothesized that a longer duration of HA use and acquisition of new HAs after 5 yrs might allow individuals to become more adept to devices and adapt to improved hearing, thereby improving communication and develop more rewarding interpersonal interactions. However, our results did not confirm this. It remains unclear how long it takes for users to benefit from HAs in various aspects of life. In the Netherlands, individuals are eligible for an HA replacement after 5 yrs. If the user is satisfied with their new devices, this may result in a new period where well-being rises shortly, then plateaus again. The 0 to 5 and 5- to 10-yr timeframes may not capture these smaller, transient fluctuations in well-being, which could explain the insignificant results. Future research would benefit from examining well-being outcomes at shorter time intervals over the long term.

### Strengths and Limitations

A major strength of this study lies in its novel design. By only selecting non-HA users with disabling hearing impairment at baseline and observing who adopted an HA over time, we created makeshift “intervention” and “control” groups while controlling for confounders, helping approach causality. To our knowledge, only one other study has used a similar design to assess HA effects (Dawes et al. 2015), but our study is the first to examine various socio-emotional well-being components separately. The 15-yr longitudinal approach is a unique aspect of our research, and by using SRTn as the measure for hearing ability, we capture real-world hearing challenges, which may better align with the expected benefits of HAs compared with pure-tone audiometry.

This study has limitations that should be considered when interpreting the results. Participants more affected by their hearing impairment might be less likely to participate, resulting in selection bias, which could lead to skewed outcome distributions. Indeed, all well-being outcomes were right-skewed, suggesting overall good well-being, which makes detecting an effect of an HA more challenging. This is particularly true for participants who already have the lowest possible baseline outcome scores, in which detecting an improvement over time is not possible. In addition, the questionnaires used may not fully capture the negative impact of hearing impairment on well-being, as negative emotions linked to hearing impairment are often fleeting and situation-specific (Holman et al. 2023). More hearing-specific situational questions, along with more frequent measurements, might better capture emotional states related to hearing impairment and HA use. Individuals with hearing impairment may also report negative feelings toward their HAs (Stark & Hickson 2004; Heffernan et al. 2016), and providing HAs without additional support or rehabilitation might not yield expected improvements (Tavanai et al. 2023). Future studies should address these factors.

Discrepancies between our study and others may arise from how “HA uptake” and “HA use” are defined. The majority of studies examine HA use rather than uptake, limiting comparability with our results. In addition, the way participants interpreted the question “Do you use a hearing aid?” may have led to misclassification of groups, as some may own an HA but not use it, resulting in “No” responses. Indeed, research has shown differences in improvements in quality of life in first-time versus more experienced HA users (Wolff et al. 2024). In addition, a “Yes” response could indicate HA use of anywhere between >0 and ≤5 yrs, creating variability between users and complicating comparability with other studies assessing HA use with the same timeframe. Moreover, nonusers do not provide information on their duration of use, so this was determined by their duration in the NL-SH. Consequently, the large study time intervals did not enable us to compare shorter durations of HA use with the same durations of nonuse. This also did not enable us to potentially capture a so-called “honeymoon effect” of HA use, representing initial improvements due to HAs in the first few months (Ivory et al. 2009). HA replacement after 5 yrs could also introduce a new cycle of adjustment and benefit, leading to temporary changes in well-being. Five-year intervals may then obscure shorter-term boosts or declines in well-being associated with the uptake of new devices.

Although this study aimed to evaluate the general effect of HAs on well-being, future research could benefit from exploring additional factors, such as HA fitting, satisfaction (e.g., quality or appearance), device-related challenges (e.g., handling and maintenance) and barriers and facilitators to consistent use, which may influence the effectiveness of HAs. These variables were not measured in the present study but could help explain variability in how HAs affect well-being across different groups. Moreover, unmeasured factors, such as cognitive function, income, and perceived social stigma, might have contributed to residual confounding and should be considered in future studies to better isolate the effects of HA use on socio-emotional well-being.

## CONCLUSION

This exploratory longitudinal study uncovered several significant findings concerning the effects of HA uptake, duration of use, and frequency of use on various socio-emotional well-being outcomes. By contributing valuable evidence to the growing body of research on the psychosocial benefits of HAs, this study highlights both the potential and the limitations of device use in improving well-being. The lack of significant findings in some areas suggests that HAs alone may not adequately address psychosocial well-being. For individuals with poorer psychosocial health, audiologists might consider integrating psychosocial support as part of a comprehensive treatment approach beyond simply recommending HA adoption or increased usage, as well-being outcomes may not solely rely on device use.

## SUMMARY OF FINDINGS

- Among individuals without tinnitus, HA uptake was significantly associated with lower depression scores (*p* < 0.05). Among those aged >65 yrs, HA uptake was significantly associated with lower total loneliness scores. No significant associations were found between HA uptake and anxiety, somatization, distress, and emotional loneliness.
- Self-perceived hearing disability did not mediate the relationship between HA uptake and socio-emotional well-being outcomes.
- No significant associations between the duration of HA use and socio-emotional well-being outcomes were found.
- Frequency of HA use was not significantly associated with any outcome except somatization, where using a HA for 1 to 4 hr per day was significantly associated with lower somatization scores.

## ACKNOWLEDGMENTS

The authors would like to acknowledge and thank the participants for their time and effort to contribute valuable data to the NL-SH.

## Supplementary Material
