# Supplementary material for: The WHAM Study: Socio-Emotional Well-being Effects of Hearing Aid Use and Mediation Through Improved Hearing Ability
Source: Ear Hear. 2025 Jul 25;46(6):1641–51. doi: 10.1097/AUD.0000000000001700 (PMC12533782; doi:10.1097/AUD.0000000000001700)
Supplement: Supplementary file 1 [file aud-46-1641-s001.pdf]

Supplementary Material (SM)

The WHAM Study:
Socio-Emotional Wellbeing Effects of Hearing Aid Use and Mediation
through Improved Hearing Ability

The supplementary material for the article titled, “Socio-emotional wellbeing effects of hearing aid use and mediation through improved hearing ability”, is composed of three parts:

SM1: Flowcharts for RQs.
SM2: Figures.
SM3: Additional Tables.

SM1

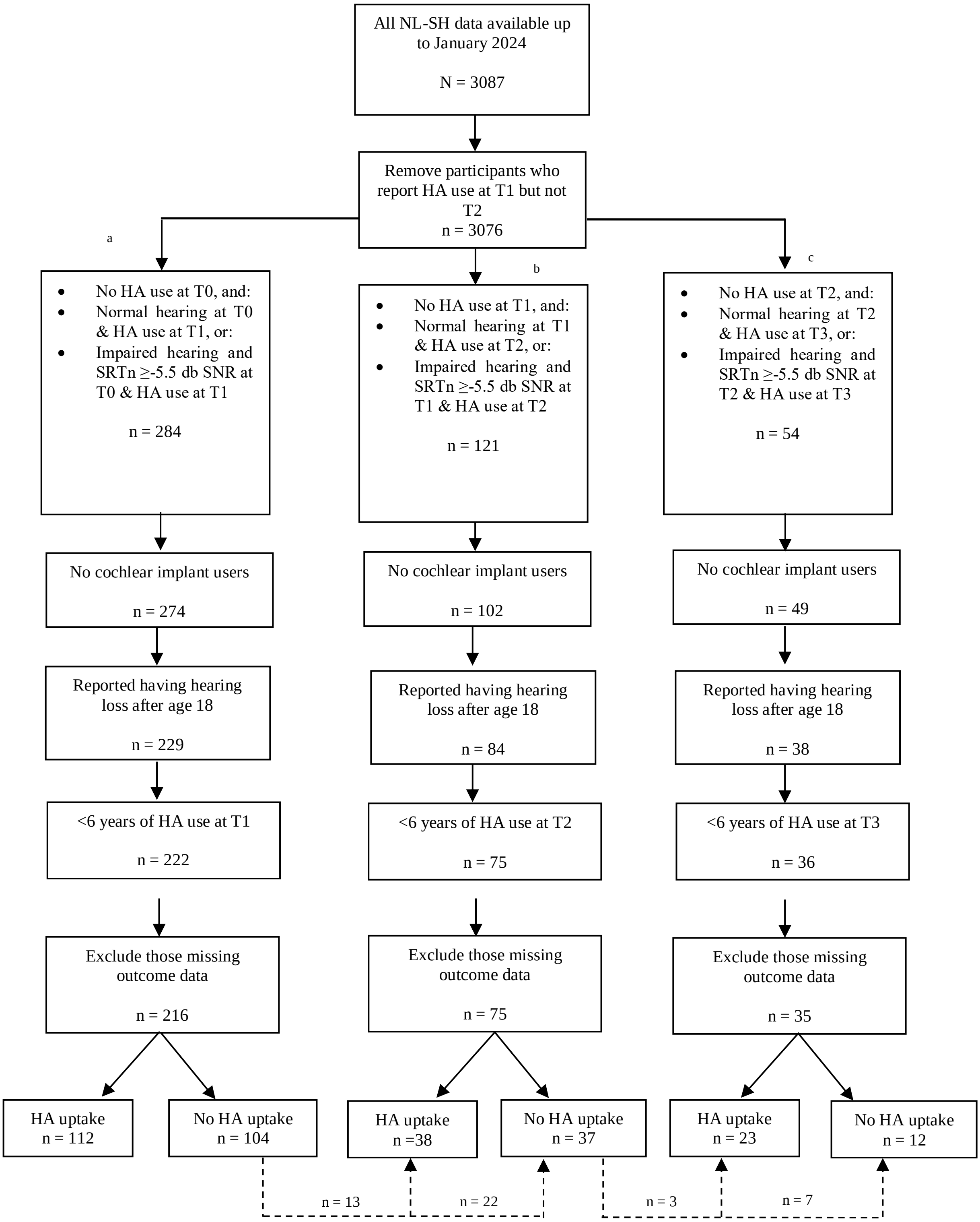

Fig. 1 Flowchart for RQ1

Note. a-, b-, and c- paths represent exclusion pathways for T0-T1, T1-T2, and T2-T3, respectively.

Abbreviations. HA, hearing aid; T0, baseline; T1, 5-year follow-up; T2, 10-year follow-up; T3, 15-year follow-up; SRTn, speech reception threshold in noise; SNR, signal-to-noise ratio.

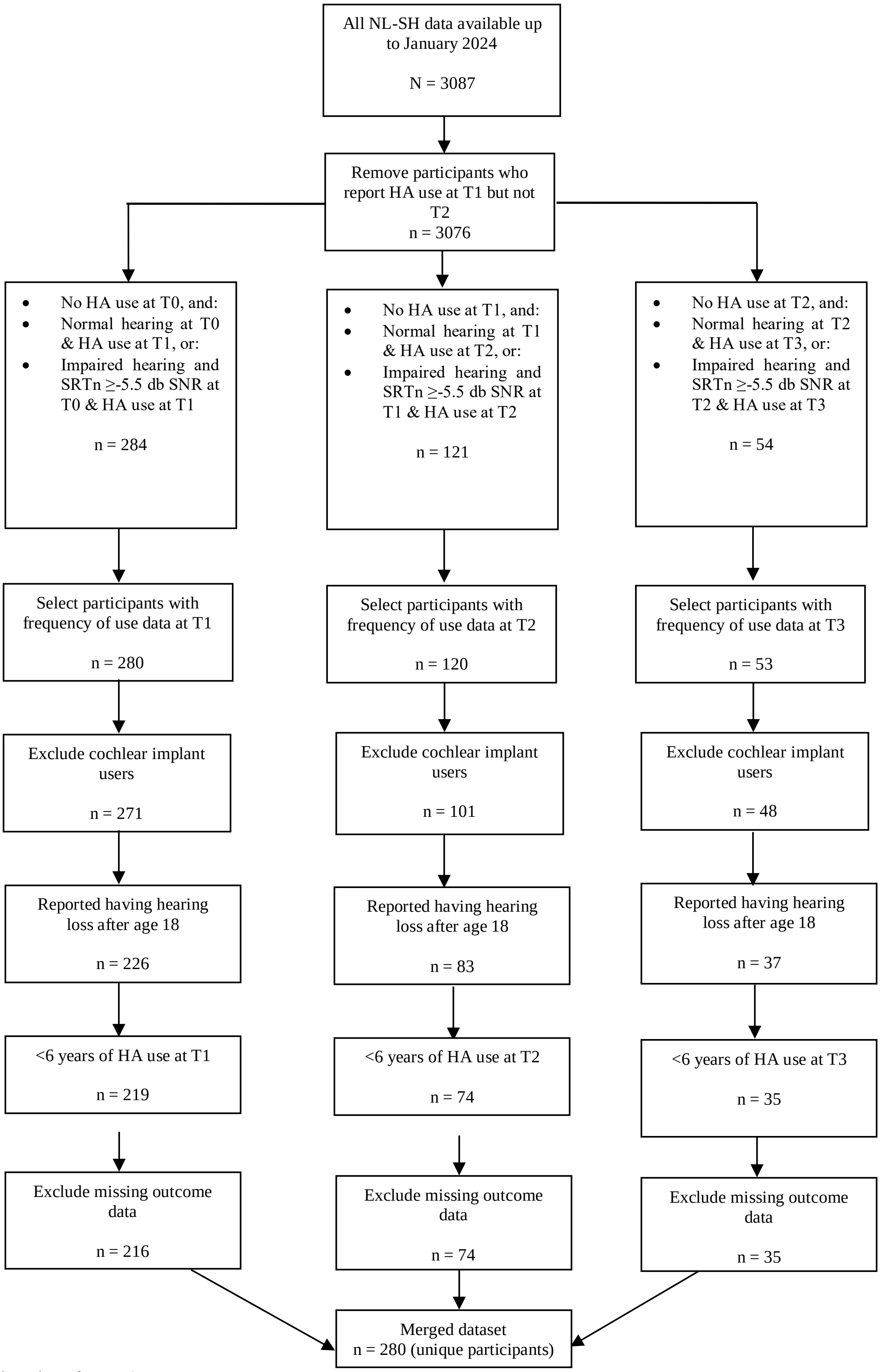

Fig. 2 Flowchart for RQ2

Abbreviations. HA, hearing aid; T0, baseline; T1, 5-year follow-up; T2, 10-year follow-up; T3, 15-year follow-up; SRTn, speech reception threshold in noise; SNR, signal-to-noise ratio.

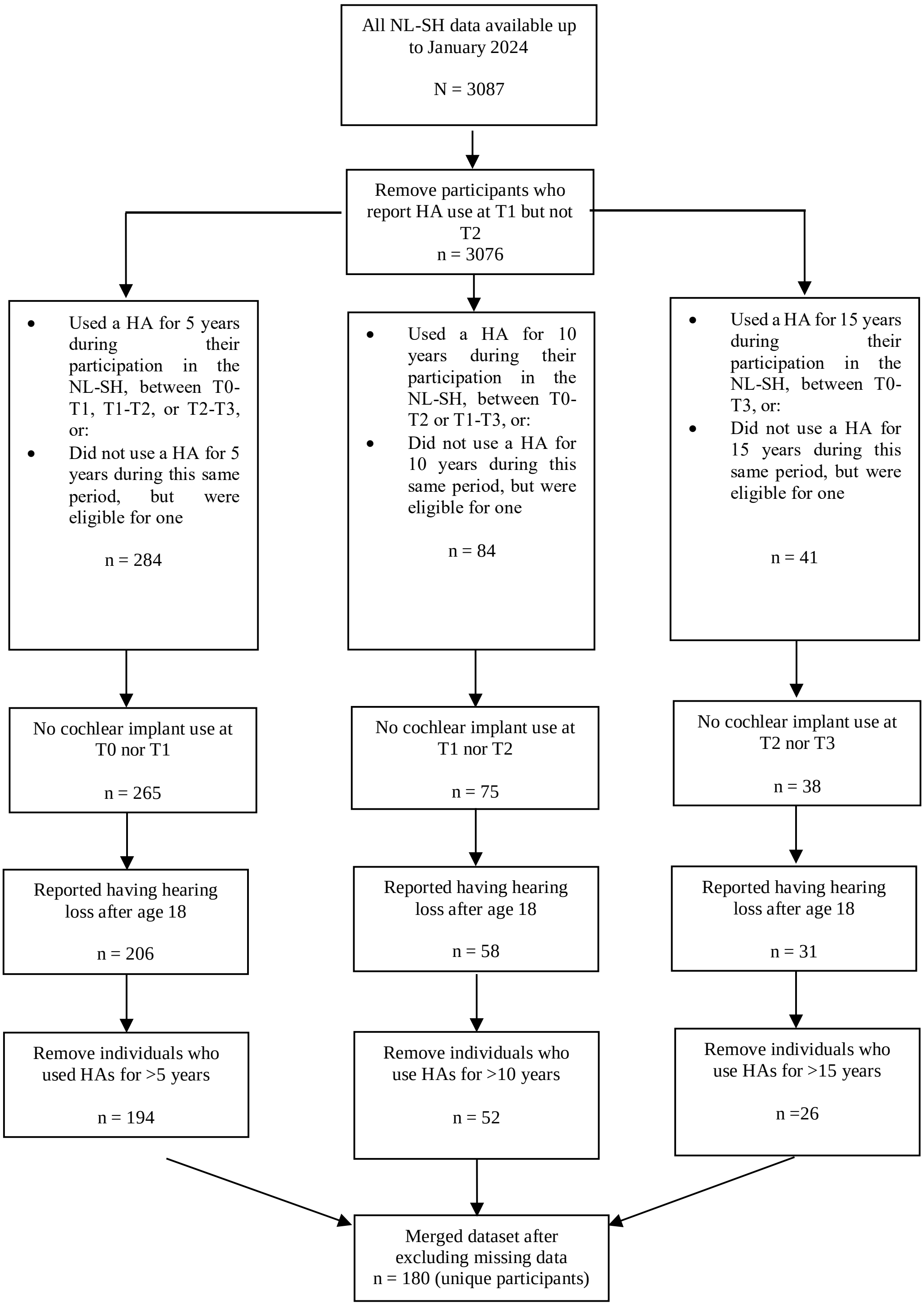

Fig. 3 Flowchart for RQ3
Abbreviations. HA, hearing aid; T0, baseline; T1, 5-year follow-up; T2, 10-year follow-up; T3, 15-year follow-up; SRTn, speech reception threshold in noise; SNR, signal-to-noise ratio.

SM2

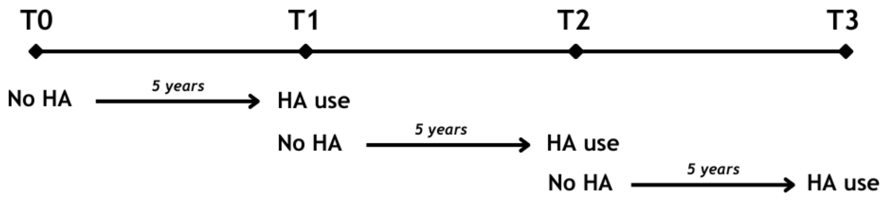

Fig. 1. Creation of hearing aid (HA) uptake independent variable for RQs 1, 1(a), and 1(b).

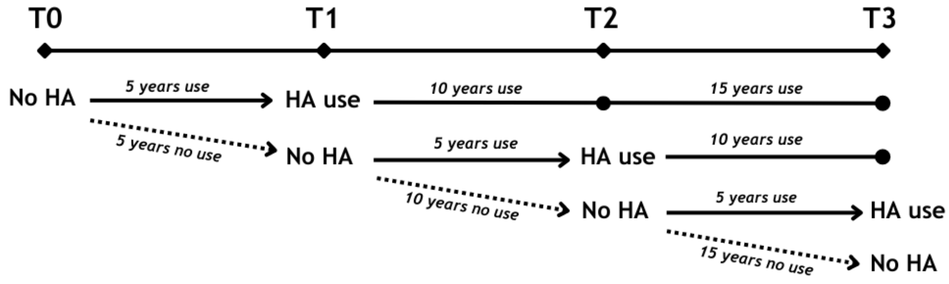

Fig. 2. Creation of duration of hearing aid (HA) use/nonuse independent variable for RQ3.

SM3

TABLE 1. Estimated marginal means (EMMs) for HA uptake and wellbeing outcomes (RQ1).

	HA uptake	No HA uptake
	M (95% CI)	M (95% CI)
Depression (0-12)		
Tinnitus	1.10 (0.70, 1.73)	0.90 (0.66, 1.23)
No tinnitus	0.43 (0.28, 0.67)*	1.42 (0.84, 2.38)*
Anxiety (0-24)		
Hyperacusis	1.86 (1.37, 2.72)	1.67 (1.20, 2.32)
No hyperacusis	0.76 (0.56, 1.04)	1.23 (0.81, 1.85)
Distress (0-32)		
Hyperacusis	10.0 (8.59, 12.0)	8.44 (7.46, 9.72)
No hyperacusis	5.71 (4.85, 6.94)	6.48 (5.30, 8.33)
Somatization (0-32)	5.90 (5.20, 6.69)	6.84 (6.04, 7.74)
Total loneliness (0-11)		
≤65 yrs	2.76 (2.37, 3.29)	2.91 (2.57, 3.56)
>65 yrs	2.86 (2.37, 3.60) *	3.05 (2.53, 3.85)
Social loneliness (0-5)		
≤65 yrs	1.93 (1.69, 2.26)	1.83 (1.59, 2.16)
>65 yrs	2.02 (1.68, 2.54)	2.02 (1.68, 2.54)
Tinnitus	2.03 (1.75, 2.42)	1.86 (1.59, 2.25)
No tinnitus	1.73 (1.44, 2.15)	2.11 (1.72, 2.72)
Emotional loneliness (0-6)	1.23 (1.03, 1.64)	1.27 (1.05, 1.60)

Note. Means and CIs shown are after transformations (+0.1) were performed for these outcomes.
Abbreviations. HA, hearing aid; M, mean; CI, confidence interval.
* Statistically significant (p<0.05)

TABLE 2. Mediation results for the relationship between HA uptake and wellbeing outcomes (RQ1(b)).

Outcome<br>*AIADH scores*	Effect of mediator on DV<br>(b [95% CI])	Effect of IV on mediator<br>(b [95% CI])	Natural direct effect<br>(b [95% CI])	Natural indirect effect<br>(b [95% CI])	*p**
Depression					
Total Score	0.19 [-0.11, 0.49]	0.22 [-0.17, 0.62]	0.07 [-0.19, 0.32]	-0.03 [-0.39, 0.42]	0.89
Intelligibility in noise	0.25 [-0.08, 0.57]	0.41 [-0.07, 0.88]	0.07 [-0.18, 0.32]	-0.03 [-0.39, 0.33]	0.86
Intelligibility in quiet	0.19 [-0.08, 0.46]	-0.01 [-0.44, 0.42]	0.05 [-0.20, 0.29]	0.00 [-0.36, 0.35]	0.98
Auditory localization	0.14 [-0.13, 0.42]	0.31 [-0.09, 0.71]	0.06 [-0.20, 0.31]	-0.02 [-0.38, 0.35]	0.93
Distinguishing sounds	0.16 [-0.12, 0.44]	0.23 [-0.24, 0.69]	0.07 [-0.19, 0.32]	-0.02 [-0.39, 0.34]	0.93
Detection of sounds	0.28 [-0.01, 0.58]	-0.06 [-0.53, 0.41]	0.03 [-0.20, 0.27]	0.01 [-0.34, 0.36]	0.96
Anxiety					
Total Score	0.22 [0.04, 0.40]†	0.22 [-0.17, 0.62]	0.04 [-0.11, 0.17]	-0.02 [-0.23, 0.19]	0.85
Intelligibility in noise	0.20 [0.03, 0.38]†	0.41 [-0.07, 0.88]	0.04 [-0.11, 0.18]	-0.04 [-0.24, 0.19]	0.81
Intelligibility in quiet	0.15 [0.00, 0.30]	-0.01 [-0.44, 0.42]	0.01 [-0.13, 0.16]	0.00 [-0.21, 0.21]	0.98
Auditory localization	0.10 [-0.07, 0.26]	0.31 [-0.09, 0.71]	0.02 [-0.13, 0.18]	-0.01 [-0.23, 0.20]	0.91
Distinguishing sounds	0.13 [-0.04, 0.29]	0.23 [-0.24, 0.69]	0.02 [-0.13, 0.17]	-0.01 [-0.32, 0.20]	0.91
Detection of sounds	0.23 [0.07, 0.39]†	-0.06 [-0.53, 0.41]	0.00 [-0.13, 0.14]	0.01 [-0.20,0.21]	0.94
Distress					
Total Score	0.02 [0.00, 0.04]†	0.22 [-0.17, 0.62]	0.00 [-0.02, 0.02]	0.00 [-0.03, 0.02]	0.86
Intelligibility in noise	0.02 [0.00, 0.00]	0.41 [-0.07, 0.88]	0.00 [-0.02, 0.02]	0.00 [-0.03, 0.02]	0.83
Intelligibility in quiet	0.02 [0.00, 0.04]†	-0.01 [-0.44, 0.42]	0.00 [-0.02, 0.02]	0.00 [-0.03, 0.02]	0.98
Auditory localization	0.01 [0.00, 0.03]	0.31 [-0.09, 0.71]	0.00 [-0.02, 0.02]	0.00 [-0.03, 0.02]	0.91
Distinguishing sounds	0.01 [0.00, 0.03]	0.23 [-0.24, 0.69]	0.00 [-0.02, 0.02]	0.00 [-0.03, 0.02]	0.87
Detection of sounds	0.02 [0.01, 0.04]†	-0.06 [-0.53, 0.41]	0.00 [-0.02, 0.02]	0.00 [-0.02, 0.02]	0.99
Somatization					
Total Score	0.03 [0.01, 0.05]†	0.22 [-0.17, 0.62]	-0.03 [-0.04, -0.01]†	0.00 [-0.03, 0.02]	0.80
Intelligibility in noise	0.04 [0.02, 0.06]†	0.41 [-0.07, 0.88]	-0.02 [-0.04, 0.00]†	-0.01 [-0.03, 0.02]	0.63
Intelligibility in quiet	0.04 [0.02, 0.05]†	-0.01 [-0.44, 0.42]	-0.03 [-0.05, -0.01]†	0.00 [-0.03, 0.03]	0.85
Auditory localization	0.02 [0.01, 0.04]†	0.31 [-0.09, 0.71]	-0.03 [-0.05, -0.01]†	0.00 [-0.03, 0.03]	0.80
Distinguishing sounds	0.02 [0.00, 0.04]†	0.23 [-0.24, 0.69]	-0.03 [-0.05, -0.01]†	0.00 [-0.03, 0.03]	0.84
Detection of sounds	0.04 [0.02, 0.05]†	-0.06 [-0.53, 0.41]	-0.03 [-0.05, -0.01]†	0.00 [-0.03, 0.03]	0.95
Total Loneliness					
Total Score	0.02 [-0.01, 0.06]	0.22 [-0.17, 0.62]	-0.04 [-0.07, 0.00]	0.00 [-0.05, 0.05]	0.93
Intelligibility in noise	0.02 [-0.01, 0.06]	0.41 [-0.07, 0.88]	-0.03 [-0.07, 0.00]	0.00 [-0.05, 0.04]	0.93

Intelligibility in quiet	0.05 [0.01, 0.08]†	-0.01 [-0.44, 0.42]	-0.04 [-0.07, 0.00]†	0.00 [-0.05, 0.05]	0.96
Auditory localization	0.00 [-0.03, 0.04]	0.31 [-0.09, 0.71]	-0.04 [-0.07, 0.00]†	0.00 [-0.05, 0.05]	0.98
Distinguishing sounds	0.02 [-0.01, 0.06]	0.23 [-0.24, 0.69]	-0.03 [-0.07, 0.00]	0.00 [-0.05, 0.04]	0.90
Detection of sounds	0.04 [0.01, 0.08]†	-0.06 [-0.53, 0.41]	-0.04 [-0.07, 0.00]†	0.00 [-0.05, 0.05]	0.96
Social Loneliness					
Total Score	0.05 [-0.02, 0.12]	0.22 [-0.17, 0.62]	-0.03 [-0.09, 0.04]	0.00 [-0.09, 0.08]	0.92
Intelligibility in noise	0.08 [0.00, 0.15]†	0.41 [-0.07, 0.88]	-0.02 [-0.08, 0.04]	-0.01 [-0.10, 0.08]	0.81
Intelligibility in quiet	0.06 [-0.01, 0.12]	-0.01 [-0.44, 0.42]	-0.03 [-0.10, 0.03]	0.00 [-0.09, 0.09]	0.95
Auditory localization	0.06 [-0.01, 0.12]	0.31 [-0.09, 0.71]	-0.02 [-0.08, 0.04]	-0.01 [-0.10, 0.08]	0.82
Distinguishing sounds	0.05 [-0.01, 0.12]	0.23 [-0.24, 0.69]	-0.02 [-0.09, 0.04]	-0.01 [-0.10, 0.08]	0.88
Detection of sounds	0.05 [-0.02, 0.11]	-0.06 [-0.53, 0.41]	-0.03 [-0.09, 0.03]	0.00 [-0.09, 0.03]	0.99
Emotional loneliness					
Total Score	0.06 [-0.03, 0.15]	0.22 [-0.17, 0.62]	-0.07 [-0.15, 0.02]	0.00 [-0.13, 0.12]	0.94
Intelligibility in noise	0.04 [-0.04, 0.13]	0.41 [-0.07, 0.88]	-0.07 [-0.16, 0.02]	0.00 [-0.13, 0.12]	0.97
Intelligibility in quiet	0.14 [0.05, 0.23]†	-0.01 [-0.44, 0.42]	-0.07 [-0.16, 0.01]	0.00 [-0.12, 0.12]	0.99
Auditory localization	-0.01 [-0.10, 0.08]	0.31 [-0.09, 0.71]	-0.07 [-0.16, 0.17]	0.00 [-0.13, 0.13]	0.99
Distinguishing sounds	0.08 [-0.01, 0.17]	0.23 [-0.24, 0.69]	-0.05 [-0.15, 0.03]	-0.01 [-0.14, 0.12]	0.88
Detection of sounds	0.13 [0.04, 0.22]†	-0.06 [-0.53, 0.41]	-0.07 [-0.16, 0.01]	0.00 [-0.12, 0.12]	0.99

Note. Mediation by self-perceived hearing disability for various listening domains was tested.
[*] *p*-value for two-sided test for mediation.
† Statistically significant ($p < 0.05$).
Abbreviations. HA, hearing aid; IV, independent variable; DV, dependent variable; b, beta coefficient; CI, confidence interval; p, *p*-value; RQ, research question.

TABLE 3. GEE results for the associations between frequency of HA use and social wellbeing outcomes.

	Total loneliness		Social loneliness		Emotional loneliness	
Frequency of HA use	b (95% CI)	*p*	b (95% CI)	*p*	b (95% CI)	*p*
1-4 hours per day	-0.11 (-0.53, 0.31)	0.61	0.03 (-0.39, 0.46)	0.88	-0.08 (-0.65, 0.50)	0.79
>4 hours per day	-0.18 (-0.39, 0.04)	0.10	-0.06 (-0.27, 0.15)	0.56	-0.14 (-0.42, 0.14)	0.33

Note. All models are adjusted for baseline outcome scores and, if indicated, additional confounders.
Abbreviations. HA, hearing aid; b, beta coefficient; CI, confidence interval; GEE, Generalized Estimated Equations; *p*, *p*-value.

TABLE 4. Estimated Marginal Means for frequency of HA use and wellbeing outcomes (RQ2).

	Non-users, 0 hours per day or >1 hour per day	1-4 hours per day	>4 hours per day
	M (95% CI)	M (95% CI)	M (95% CI)
Depression (0-12)	0.93 (0.72, 1.20)	1.12 (0.53, 2.42)	0.89 (0.57, 1.39)
Anxiety (0-24)	1.43 (1.12, 1.82)	1.10 (0.70, 1.73)	1.29 (0.96, 1.73)
Distress (0-32)	8.44 (7.58, 9.41)	7.50 (5.32, 10.6)	8.00 (6.84, 9.35)
Somatization (0-32)	7.01 (6.27, 7.84)	4.80 (3.60, 6.40)*	5.85 (5.04, 6.80)
Total loneliness (0-11)	3.56 (3.09, 4.11)	3.20 (2.15, 4.74)	2.98 (2.55, 3.50)
Social loneliness (0-5)	2.15 (1.89, 2.44)	2.22 (1.50, 3.30)	2.02 (1.73, 2.36)
Emotional loneliness (0-6)	1.55 (1.27, 1.88)	1.43 (0.84, 2.44)	1.35 (1.08, 1.67)

Note. Means and CIs shown are after transformations (+0.1) were performed for these outcomes.
Abbreviations. M, mean; CI, confidence interval.
* Statistically significant (p<0.05)

TABLE 5. GEE results for the associations between duration of HA use/nonuse and emotional wellbeing outcomes.

	Depression*		Anxiety		Distress		Somatization†	
HA use *time interaction	b (95% CI)	*p*	b (95% CI)	*p*	b (95% CI)	*p*	b (95% CI)	*p*
Yes * 10 years	0.06 (-0.18, 0.30)	0.634	0.56 (-0.21, 1.34)	0.155	-0.01 (-0.06, 0.04)	0.723	0.33 (-0.07, 0.72)	0.107

Note. All models are adjusted for baseline outcome scores and, if indicated, additional confounders.

*Additionally adjusted for age group.

† Additionally adjusted for health status.

Abbreviations. HA, hearing aid; b, beta coefficient; CI, confidence interval; GEE, Generalized Estimated Equations; *p*, *p*-value.

TABLE 6. GEE results for the associations between duration of HA use/nonuse and social wellbeing outcomes.

	Total loneliness		Social loneliness		Emotional loneliness	
HA use *time interaction	b (95% CI)	*p*	b (95% CI)	*p*	b (95% CI)	*p*
Yes * 10 years	-0.32 (-0.86, 0.23)	0.258	-0.18 (-0.74, 0.39)	0.539	-0.40 (-1.15, 0.35)	0.294

Note. All models are adjusted for baseline outcome scores and, if indicated, additional confounders.

Abbreviations. HA, hearing aid; b, beta coefficient; CI, confidence interval; GEE, Generalized Estimated Equations; *p*, *p*-value.

TABLE 7. Estimated Marginal Means for duration of HA use and wellbeing outcomes (RQ3).

	HA use M (95% CI)		No HA use M (95% CI)	
	5 years	10 years	5 years	10 years
Depression (0-12)	0.62 (0.53, 0.72)	0.64 (0.55, 0.74)	0.58 (0.51, 0.67)	0.57 (0.45, 0.72)
Anxiety (0-24)	1.07 (0.78, 1.46)	1.07 (0.76, 1.51)	1.45 (1.08, 1.95)	0.83 (0.46, 1.52)
Distress (0-32)	6.74 (5.88, 7.90)	7.25 (5.98, 9.20)	7.23 (6.37, 8.36)	7.33 (5.94, 9.57)
Somatization (0-32)	5.59 (4.87, 6.41)	6.36 (5.31, 7.61)	7.26 (6.29, 8.39)	5.96 (4.35, 8.17)
Total loneliness (0-11)	2.90 (2.43, 3.47)	2.69 (2.03, 3.56)	3.13 (2.57, 3.82)	3.97 (2.63, 5.98)
Social loneliness (0-5)	1.88 (1.59, 2.23)	1.95 (1.46, 2.59)	2.06 (1.70, 2.50)	2.55 (1.69, 3.84)
Emotional loneliness (0-6)	1.30 (1.00, 1.69)	1.08 (0.74, 1.58)	1.43 (1.11, 1.84)	1.77 (1.01, 3.10)

Note. Means and CIs shown are after transformations (+0.1) were performed for these outcomes.
